# Impact of electric vechicles on power transmission grids

**DOI:** 10.1016/j.heliyon.2023.e22253

**Published:** 2023-11-13

**Authors:** Gustavo Adolfo Gómez-Ramírez, Rebeca Solis-Ortega, Luis Alberto Ross-Lépiz

**Affiliations:** a Electromechanical Engineering School, Costa Rica Institute of Technology, Cartago, 3010, Costa Rica; b Mathematics School, Costa Rica Institute of Technology, Cartago, 3010, Costa Rica

**Keywords:** Electric vehicles, Forecasting methods, Power planning, Power system management

## Abstract

This paper presents a methodology for assessing the impact of electric vehicles (EVs) on the power transmission grid of the Costa Rica Power System. The methodology considers penetration scenarios, user preferences, charging habits, and expected fleet growth. Using ETAP software, the study simulates power flow, demand behavior, and voltage levels in the presence of high penetration of electric vehicles. The analysis covers a 15-year horizon and focuses on voltage and demand profiles in 2025, 2030, and 2040. The results indicate a decline in voltage profiles that reaches dangerous levels after 2030, primarily in the distribution grid, and an increase in demand by  for 2040 in the most severe scenario. The analysis also reveals several key findings (a) the identification of problems in the electrical infrastructure starting in 2030 and a major insufficiency in accommodating the increase in EVs by 2040; (b) the need to evaluate stability in transmission grids considering loadability and voltage; (c) the necessity of investing in electrical infrastructure, driven by public policies, to meet future energy requirements and strengthen transmission networks; (d) the significance of accounting for both EV growth and electric infrastructure improvements in system analysis; and (e) the anticipation that the system's performance will fall within the extreme demand values presented in the analysis. The study emphasizes the importance of considering a broader range of scenarios and variability in parameters, especially user charging behaviors, to enable decision-makers to plan for the challenges and opportunities associated with the widespread adoption of EVs in a country's power grid.

## 1 Introduction

The presence of electric vehicles (EVs) has grown significantly worldwide in the past decade. In 2012, approximately 120,000 electric cars were sold globally. By 2021, this number had surged to 6.6 million, accounting for 10% of global car sales. Notably, global EV adoption reached about 16.5 million vehicles in 2021, tripling the figure from 2018. Sales in the first quarter of 2022 have already increased by 75% compared to 2021 [1].

The surge in electric vehicle adoption can be attributed to two primary drivers: environmental concerns and government initiatives. EVs are appreciated for their eco-friendly characteristics, including low emissions, reduced indirect emissions concentrated at power stations, fuel independence, and decreased noise pollution, as documented by Nour et al. [2].

Government policies promoting electric vehicle acquisition have played a crucial role, with several countries offering incentives such as purchase subsidies, tax benefits, and more [2]. Xue et al. [3] investigated these policies in 20 countries, revealing that they contribute to increased EV adoption. Tax reduction policies, charger density, and income have the most significant impact.

Despite the many advantages of electric vehicles, a key concern is whether power systems can handle the increased load from a large number of EVs charging simultaneously [1]. Various studies have explored this issue, including research by Szabłowski and Bralewski [4] in Poland, Suski et al. [5] in the Maldives, Almohaimeedal [6] in Saudi Arabia, Betancur et al. [7] in Colombia, Strobel et al. [8] in Germany, and Di Chiara et al. [9] in Uruguay. These studies employed various methodologies and considered different variables and scenarios.

This paper presents a comprehensive methodology for assessing the impact of Electric Vehicles (EVs) on the Costa Rica Power System's transmission grid. The study analyzes various factors, including penetration scenarios, user preferences, charging habits, and fleet growth projections. Simulations using ETAP software assess power flow, demand behavior, and voltage levels with high EV penetration. The study covers 15 years, focusing on 2025, 2030, and 2040. By exploring the implications of EV integration, this research informs power system planning and infrastructure development and contributes a reproducible methodology for analyzing EV impacts on power systems.

The structure of the paper is as follows: Section 2 presents the methodology, Section 3 offers a case study of Costa Rica, divided into four sub-sections, Section 4 details the modeling and simulations, Section 5 presents the results and discussion, and Section 6 concludes the paper.

## 2 Methodology

Performing an EVs impact analysis on a region's power systems is a complex task. This is due to the fact that many variables can or should be taken into account. Some of these variables are (i) Power system features, (ii) Quantity of electric vehicles, (iii) EVs features, (iv) Charging habits, which include charging time slots, battery charging modes, and others.

**Figure 1:**
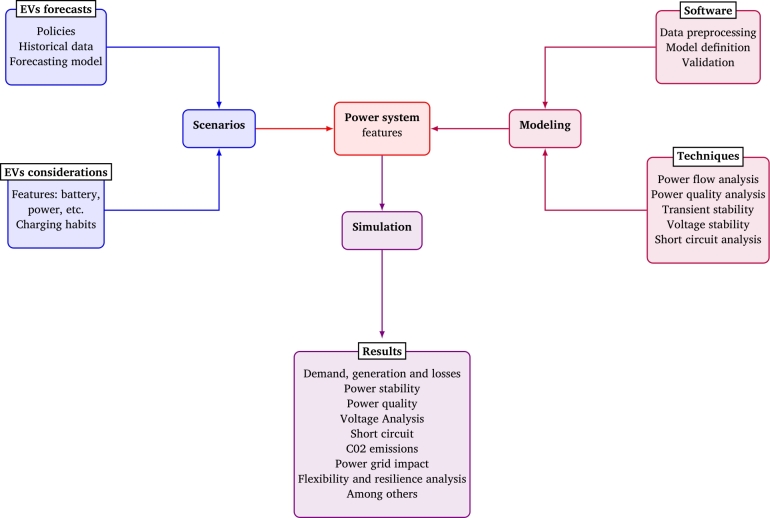
Methodology for analyzing EVs impact on a region's power system.

To address this situation, the methodology shown in Fig. 1 is proposed and summarized as follows. The first two tasks are scenarios and modeling, which may be performed independently.

In scenarios, the combinations of variables to be analyzed will be defined. We have divided them into two groups: EV forecast and EV considerations. The first focus on predicting how many electric vehicles can be available at a given time and place. It considers several factors that can affect this analysis, such as regional policies, historical data, forecasting models, and others. The second includes variables related to vehicle specifications, people's charging habits, and other related variables.

On the other hand, modeling is a recursive process that will depend on the selected software and analysis techniques. Software must integrate everyone the power elements and devices. To build a scenario it is necessary to have data on generation and demand. For this reason, data management gives the behavior profile scenario for a specific day. Therefore, the model and simulation must consider all elements of power transmission.

Information on power transformers, generators, loads, transmission lines, and reactive power compensation, among others, must be integrated into the model. For this reason, software capacities (bus quantity) become an aspect to consider, and to choose the modeling to be carried out is decided. The validation is done by considering real measurements and comparing them with the simulation results. When those processes are well-defined, they must be joined with the power system features to proceed with the simulation.

Once the power grid model is built, it can be obtained: *power flows between the lines, generation dispatch, demand, loadability, among others*. Data processing results can obtain an analysis of stability, power quality, flexibility, and resilience, short-circuit analysis among others, and analyze voltage, demand behavior, and $CO_{2}$ emission. The final stage consists of analyzing these findings. This methodology was used to analyze the impact of electric vehicles on the Costa Rican power system. Each of the stages, considered variables, and the results are explained below.

## 3 Study case: Costa Rica

In this section, we will delve into a study case of the impact of electric vehicles on Costa Rica's power system. We will include three key components, which are: Power system description, EVs data and country's policies, and EV forecast.

The first subsection, Power system description, will provide an overview of the current power system infrastructure in Costa Rica. This will serve as a backdrop for the rest of the analysis in the following sections.

The second subsection, EVs data and country's policies, will examine the current state of EVs in Costa Rica, including the number of EVs on the road, their characteristics, and the government policies to promote their use.

Finally, the EV forecast subsection will use available data and a forecasting method to predict the future growth of EVs in Costa Rica.

### 3.1 Power system description

**Figure 2:**
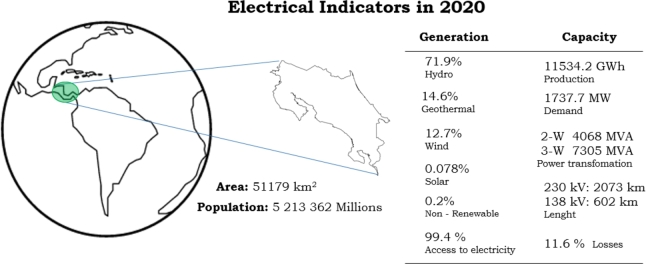
Costa Rica power system description based on [14].

Costa Rica is located in Central America, its surface is , and has a population of 5,213,362 inhabitants. The access to electricity is 99.4% with a per capita consumption of /inhabitant in 2020. SEN (*Spanish acronym for the National Electric System*), is made up of a large number of renewable generation sources where the predominant generation resource is the Hydraulic followed by Geothermal as shown in Fig. 2.

The installed capacity for generation [10] is the 66% by Hydroelectric resources, 13% in Non-Renewable resources, 12% in Wind resources, 7% in Geothermal resources, 2% in Biomass generation, and 0.1% in PV resources. Transmission Power Grid has a voltage level of 138 and  while the distribution system is in 69, 34.5 and  [10], [11]. Power losses in the grid were 11.6% in 2020. Costa Rica has 5 International Interconnections with Nicaragua and Panamá.

Costa Rica also has 2 transmission interconnections with Nicaragua, and 3 with Panama. Those transmission interconnections are of  [10], [11]. The Power Transformation installed is . In Two Winding (2-W) has  and in Three Winding (3-W) has  according to Fig. 2. In Table 1 the demand forecasting is shown in 2025, 2030, and 2040. Power System has a huge capacity for integrating intermittent renewable resources and the power grid support it [12]. Nevertheless, it must take advantage of distributed resources [13] as PV Generation to supply the power demand for electric vehicles.

**Table 1:** Costa Rica power demand forecasts base on [10].

Year	Low (MW)	Base (MW)	High (MW)
2025	1767	1824	1856
2030	1835	1990	2070
2040	1964	2271	2436

### 3.2 EVs data and country's policies

EVs were introduced to the Costa Rican grid a few years ago. There are records of how many vehicles have come into the country since 2011 [15]. Table 2 presents historical data for the cumulative number of EVs in the country.

**Table 2:** Cumulative number of EVs in Costa Rica. Based on [15].

Year	2011	2012	2013	2014	2015	2016	2017	2018	2019	2020	2021
EVs	102	114	146	159	163	175	200	398	857	1484	2529

Data presented in Table 2, shows that EVs incursion happened initially at a slow pace. It was not until 2017 that EV acquisitions started to increase. This was due to the entry into force of Law 9518 on Incentives and Promotion for Electric Transportation. This bill creates incentives for EV buyers. Some of these are tax exoneration, circulation priority, easy credit access, and low-interest rates [16].

Currently, there are 20 electric vehicle brands comprising 37 models. All of them have different features. In [15] the main characteristics of these vehicles can be reviewed and are summarized in Table 3. For current models, their battery capacity spans from , with Renault Kangoo Z.E., to , with Audi E-Tron S and E-Tron . The average is . Power range from , with Renault Kangoo Z.E., to , with BYD Tang. The average is  Autonomy goes from , with JMC N801, to , with BYD Han. The average is .

**Table 3:** EV features. Based on [15].

Feature	Lower bound	Higher bound	Average
Battery capacity			
Power			
Autonomy			

For this research, historical data was used as input for the forecasting models. And among EV features, we only considered battery capacity.

### 3.3 EV forecast

Forecasting EVs sales is necessary to plan changes or upgrades to power grids and provide basic facilities such as charging stations and others [17].

Several models and approaches have been developed for predicting the adoption of electric vehicles [17], [18], [19], [20]. Wu and Chen classify these models into two main categories: statistical and machine learning techniques [17]. Univariate models focus on analyzing changes in a single variable, such as the annual number of new vehicles. In contrast, multivariable models take into account various factors, including policies, regulations, oil price fluctuations, and electricity costs, to assess their impact on EV penetration [19].

Although multivariate models, as highlighted by Zhang [19], can provide more comprehensive and accurate forecasts, their practical application may encounter challenges due to limited data availability, which can vary depending on the research location. Therefore, the selection of the most suitable model for each study heavily depends on the quantity, quality, and type of data accessible, ensuring precise predictions.

In the context of Costa Rica, as elaborated in section 3.2, the available data on the annual entry of electric vehicles into the country is scarce, consisting of only 12 historical records. Furthermore, considering that Law 9518 came into effect only in 2017, the dataset is further limited to just six available data points. Additionally, due to public policies safeguarding personal data, obtaining precise information regarding the vehicles, individuals, and specific areas they traverse is not feasible.

Due to these limitations, the current study employed a univariate methodology that specifically considers the annual number of new vehicles entering the country. As a result, the Gompertz mathematical model was chosen for analyzing the time series.

Initially developed by Benjamin Gompertz for tumor growth prediction in the medical field [21], the Gompertz model has shown its efficacy in various domains, including the forecasting of electric vehicle numbers [18], [22], [23]. Its general mathematical model is as follows:

(1) $$Y_{t}=M\cdot e^{-\alpha \cdot e^{-\beta t}}.$$

Where, $Y_{t}$ is the cumulative number of adoptions at time *t*, *M* is the number of potential adopters and *α* and *β* are parameters to be estimated by fitting them to the observed number of cumulative adoptions.

Hence, in order to determine the theoretical maximum or upper limit (*M*) of electric vehicle adoption within a specific timeframe, the parameters *α* and *β*, which best align with the historical data of the cumulative number of electric vehicles $Y_{t}$, need to be calculated. To accomplish this, a nonlinear least squares optimization algorithm is utilized to fit the Gompertz curve to the available data.

**Figure 3:**
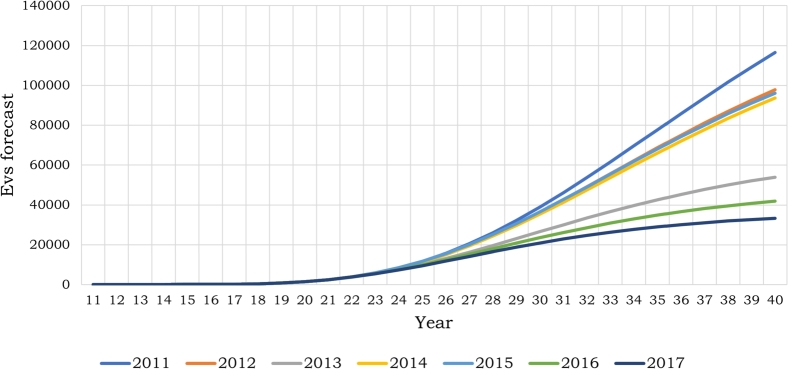
EVs forecasting curves with Gompertz model (taking data since 2011).

The study involved the calculation of seven potential Gompertz curves. Various historical datasets were utilized, covering the period from 2011 to the present, with yearly intervals. These datasets comprised the complete historical cumulative data and subsets starting from 2012, 2013, and so forth, until 2017. Fig. 3 illustrates these curves.

The analysis yielded a wide spectrum of possible EV penetration rates in Costa Rica for the forthcoming years. It becomes apparent that as the analysis expands over a longer time span, the data exhibit higher variability. This variability primarily results from the limited data and information available when utilizing this method. Nevertheless, it establishes a groundwork for examining diverse plausible scenarios.

Based on the results obtained, three curves were selected, each representing a distinct approach: one conservative, one intermediate, and one optimistic. These curves were employed to project the expected number of electric vehicles within the country for the years 2025, 2030, and 2040. The projection results are presented in Table 4.

**Table 4:** Costa Rica EV forecast.

Year	Lower bound	Middle bound	Higher bound
2025	9576	11438	11767
2030	20871	35309	38847
2040	33360	93657	116441

## 4 Modeling and simulation

### 4.1 Software selection

**Figure 4:**
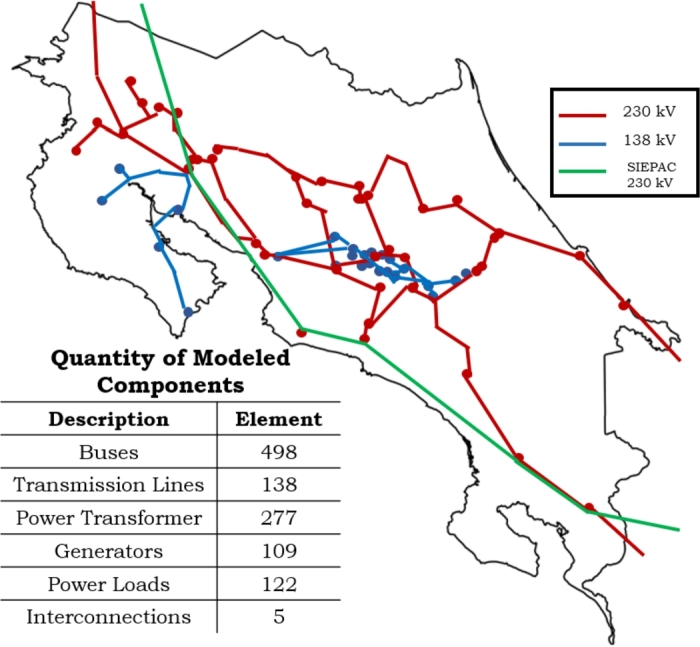
Costa Rica power system modeled using ETAP.

Costa Rica Power System was modeled and simulated using the Electrical Transient Analyser Program (ETAP) and it is an engineering design and analysis software for load flow analysis, transient, voltage stability, and short-circuit studies [24]. Fig. 4 shows the elements modeled and simulated using ETAP. The model includes current aspects for generation (Renewable and Non-Renewable), loads, power transformers, transmission lines, and reactive power compensation, among others.

Power system analysis software is essential when analyzing large power grid *p.e.* power stability of transitory and voltage analysis. It can be studied in different scenarios, for instance with EVs, storage, and renewable generation. Table 5 shows a summary of software packages used, power analysis, and several models in power system studies with EVs penetration and its corresponding paper.

**Table 5:** Power analysis, models and software package used summary.

Analyzed Techniques	Power System Models and Paper	Analysis Software Packages and Paper
Loadability	IEEE-Models [25], [26], [27], [28], [29], [30], Nordic System [31]	ETAP [18], [32], [33], [34], [35], Eurostag [36], [37], PSS/E [25], [26], [27], [28], OpenDSS [30], DIgSILENT [38]

Power Stability Frequency Regulation Control	Local Grids [27], [36], [37], Denmark [26], Spanish [39], Northern Ireland [40], IEEE-Models [30], [41], [42], [43], [44], [45], Simple Model [43], [44], Smart Grid [41], [43]	ETAP [46], DIgSILENT [26], [38], Eurostag [37], Real-Time Simulator [45], MATLAB [29], [30], [36], [43], OpenDSS [30]

### 4.2 Power transmission model

Costa Rican Power System voltage levels are 230 and  in power transmission and 34.5, 24.9 and  in distribution system as mentioned in section 3. Fig. 4 shows the configuration of the Costa Rica transmission system and the biggest transmission grid is . However, the greater concentration of electrical consumers is in the central zone of Costa Rica where the  power grid is predominated.

The SIEPAC line is the transmission line of the Regional Electricity Market and is interconnected with 5 power sub-stations inside Costa Rican Power System. The  transmission grid is interconnected (in the central region) with the  transmission power grid through the use of auto-transformers in 4 power sub-stations.

### 4.3 Scenario configuration

Based on the methodology outlined in section 2 and the case presented in section 3, a configuration was established to analyze power flow (24-hour profiles) and study power system loadability. Each scenario took into consideration:

1. *EV Battery Capacity:* As stated previously, the battery capacity of electric vehicles available in Costa Rica ranges from  to  and has an average of . So for the scenarios, these three capacity values were considered.
2. *Charging speed:* Accordingly, with [47], the charging speed can be classified into three categories:
  • Slow charging. This method uses a single-phase AC outlet of  and up to , which made it possible to fully charged a conventional electric car in 6-8 hours.
  • Fast charging. A single-phase or three-phase AC outlet with a current of up to  is used in this method. It takes between 1 to 2 hours to fully charge a conventional vehicle.
  • Rapid charging. With this option, a car can be fully charged in 5-30 minutes and uses direct current up to  between  and .
  The analyzed scenarios considered the three types of charging speeds mentioned above. For simplicity, the time duration for these types of charging were set to 7 hours, 2 hours, and 0.5 hours, respectively.
3. *Year forecast:* As stated above, this research will analyze the years 2025, 2030, and 2040. For each year, three potential scenarios for the projected amount of electric vehicles (see Table 4) will be analyzed.

In this analysis, a specific scenario is considered, taking into account the preferences and charging habits of the Costa Rican population. The electricity consumption pattern in Costa Rica shows a significant spike between 11:00 a.m. and 1:00 p.m., as well as from 5:00 p.m. to 9:00 p.m. Conversely, there is an excess of available energy during the evening. It is anticipated that approximately 50% of users will opt for fast charging due to the wide distribution of fast charging points strategically placed in various locations, including shopping centers, public and private parking lots, and areas with high population density [48].

**Figure 5:**
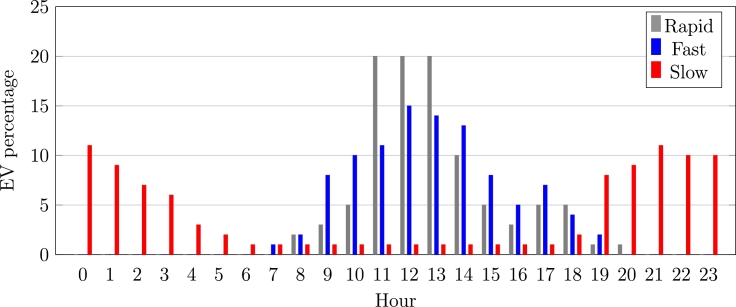
Analyzed charging speed time slot.

Similarly, this study takes into consideration individuals who choose to charge their vehicles at fast charging stations situated in public places, workplaces, or residential areas during their working hours. Moreover, users who have access to slow charging points at their homes prefer to charge their vehicles during nighttime hours. In all scenarios examined, a State of Charge (SoC) between 20% and 30% is assumed. It is worth noting that this research exclusively focuses on the specific case where 50% of vehicles opt for rapid charging, 25% select fast charging, and 25% prefer slow charging. The distribution of these charging patterns throughout the 24-hour period is illustrated in Fig. 5.

After considering all possible combinations of the parameters mentioned above, a total of 30 scenarios were analyzed.

## 5 Results and discussion

The analysis considers the winter period when there is a greater generation of hydraulic resources. The generation is concentrated in the eastern zone, however, in the summer power flows change because the generation comes from hydraulic resources from the northern zone.

In this article, it was analyzed the behavior of voltage and demand response. Voltage analysis is focused on the transmission and distribution system. Due to the impact on the power grid, it shows only the voltage results in High Demand (H). Demand Respond results are focused on the loadability-increasing behavior from Electric Vehicles Battery penetration.

### 5.1 Power demand analysis

Power flow analysis is made considering a 24-hour profile. Scenarios that contemplate the penetration of Electric Vehicles in the years 2025, 2030, and 2040 were analyzed and they are shown in Table 6. Table 6 shows the demand increase is due to the rise in capacities of batteries in Electric Vehicles and it goes from  (33-H) to  (90-H).

**Table 6:** Maximum demand reached and capacity increase in MW.

Scene analyzed	Demand			Increase		
	2025	2030	2040	2025	2030	2040
BASE	1788.7	1953.3	2254.8	**—**	**—**	**—**

33-H	1837.3	2123.9	2775.0	48.6	170.6	520.2
33-M	1827.4	2108.0	2775.0	47.1	154.7	520.2
33-L	1835.8	2043.0	2400.7	38.7	89.7	145.9

60-H	1880.6	2267.0	**—**	91.9	313.7	**—**
60-M	1877.9	2238.0	**—**	89.2	284.7	**—**
60-L	1862.7	2119.9	2523.7	74.0	166.6	268.9

90-H	1936.8	2452.5	**—**	148.1	499.2	**—**
90-M	1932.5	2406.6	**—**	143.8	453.3	**—**
90-L	1908.4	2219.5	2683.1	119.7	266.2	428.3

This table shows the results of scenarios development of the load flow analysis (demand response) and in addition, it shows the demand forecast for the years 2025, 2030, and 2040 for High (H), Medium (M), and Low (L) demand conditions.

In year 2040, for cases *60-M*, *60-H*, *90-H* and *90-M* the results show an overload condition according to Table 6. The power grid can not support the increased demand. Increasing on demand is achieved in *30-H case* () and in *90-L case* the increase is . Percent increasing penetrations is 29.9% taking into account the actual conditions of the power system for *30-h case*.

### 5.2 Voltage analysis

**Figure 6:**
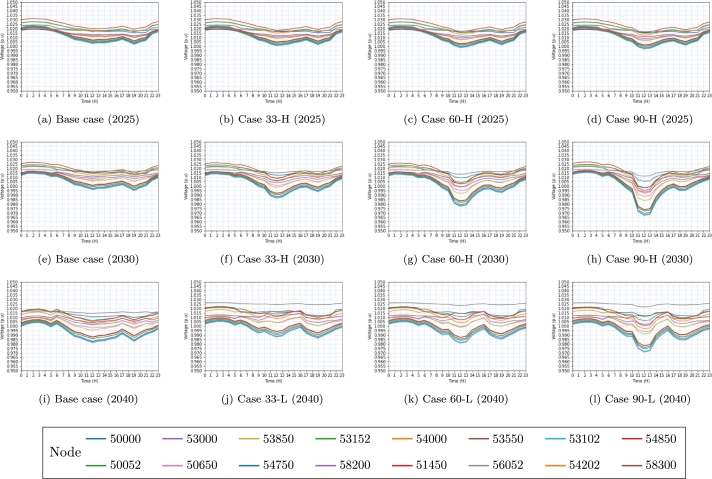
Voltage behavior on the  side in 2025, 2030, and 2040 scenarios. In each simulation, the same connection points are displayed by the identical node colors in the subfigures.

**Figure 7:**
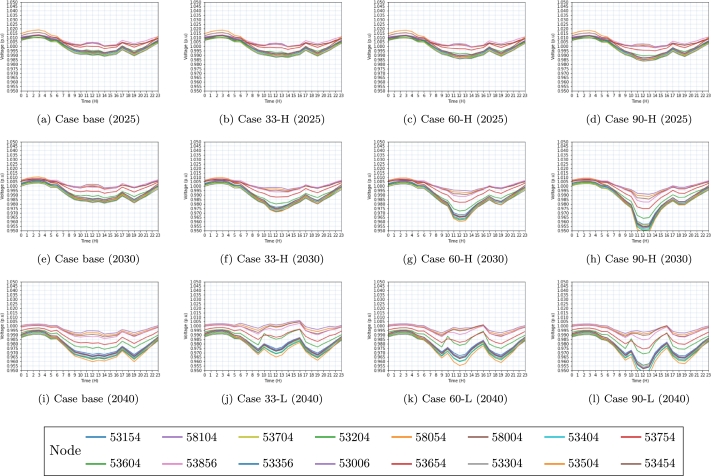
Voltage behavior on the  side in 2025, 2030 and 2040 scenarios. In each simulation, the same connection points are displayed by the identical node colors in the subfigures.

**Figure 8:**
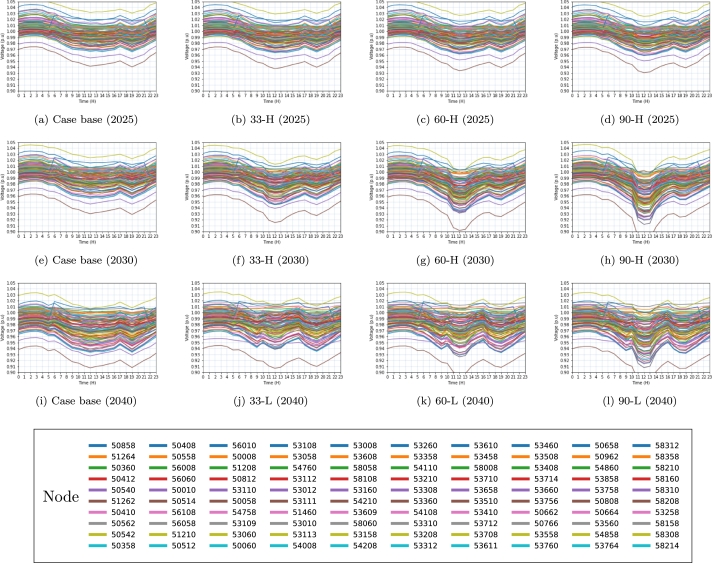
Voltage (Distribution side) behavior in 2025, 2030 and 2040 scenarios. In each simulation, the same connection points are displayed by the identical node colors in the subfigures.

The results obtained at various voltage levels evaluated in Table 6 are depicted in Figure 6, Figure 7, Figure 8. The colors assigned to each figure represent the voltage behavior observed in each electrical substation (node) throughout the integration of the EVs into the power grid.

Figure 6, Figure 7, depict a total of 16 interconnection nodes, specifically electrical substations, which have been identified as the most prominent among the nodes examined within the transmission network at both the  and  voltage levels.

The aforementioned nodes serve as critical substations within the electricity grid. In contrast, Fig. 8 shows the presence of 100 load nodes within the distribution network, namely located at the substation on the ,  and  side.

Base cases investigated are denoted in Figs. 6a, 6e, 6i, 7a, 7e, 7i, 8a, 8e, 8i. These figures represent the analysis conducted for different years, where (a) represents the year 2025, (e) represents the year 2030, and (i) represents the year 2040.

The baseline scenario does not take into account the huge penetration of EVs in the years 2025, 2030, and 2040. Without preventative measures, the base scenario is expected to show reduced voltage levels, as seen in the 2040 projections.

Figs. 6b, 6f, 6j and 7b, 7f do not exhibit evidence of low voltage issues, despite the load capacity of case 33. However, Figs. 7j and 8b, 8f, 8j demonstrate a gradual decline in voltage levels as a result of increased loadability. The reason for this change can be attributed to the significant rise in the load, which has increased from  to  in the case of 33-H, as indicated in Table 6.

Similarly, it can be observed that Figs. 6c, 6g, 6k and 7c, 7d do not depict instances of low voltage issues within the examined scenarios. The transmission network operating at  consistently maintains values within the expected parameters, as depicted in Figs. 6d, 6h, 6l.

Nevertheless, the figures depicting the transmission network at the  side reveal voltage levels that are below 0.9*pu* (per unit) as illustrated in Figs. 7g, 7h, 7i, 7j, 7k, 7l. The reason for this is that, as a result of the network structure, there exists a significant aggregation of loads that are interconnected at this specific voltage level. Likewise, Figs. 8c, 8g, 8k extracted from the dataset of 2025 exhibit instances of low voltage issues observed within certain nodes, featured by values below 0.97pu (per unit).

Based on the data presented in Table 6, it is evident that both the load capacities of 60-H and 60-M surpass the threshold of . Consequently, the scenarios examined for the year 2040 encounter convergence issues, thereby indicating that the electrical system lacks the capability to accommodate the anticipated rise in vehicle penetration. Due to this rationale, the study incorporates cases 60-L (Fig. 8k) and 90-L (Fig. 8l) instead of examples 60-H and 90-H. Fig. 8d, 8h, 8k, 8l exhibits voltage level issues (V<0.97*pu*) resulting from the substantial increase in vehicle penetration.

### 5.3 Impact emission analysis

To estimate the annual TON-equivalent of CO2 emissions, the methodology proposed in [49] was utilized. The projected emissions resulting from the penetration of vehicles are provided for the years 2025, 2030, and 2040 in Table 7. Numerous institutions rely on this methodology to calculate emissions, taking into account the unique conditions of Costa Rica.

**Table 7:** *CO*_2_-equivalent results.

Scenario	Electric Vehicles Forecast			TON per year		
	2025	2030	2040	2025	2030	2040
Low	9576	20871	33360	13480	29379	46960
Medium	11438	35309	93657	16101	49703	131841
High	11767	38847	116441	16564	54683	163914

According to the PNE (*Spanish acronym for the National Energy Plan 2015-2030*) [50], the transportation sector has 66% of hydrocarbon consumption. It produces the 54% of $CO_{2}$ emissions. Nowadays, vehicles have an average age of 15.95 years. For this reason, Costa Rica has a commitment to carbon neutrality. Table 7 shows the $CO_{2}$ equivalent in 2025, 2030, and 2040 forecast in low, medium, and high penetration as described in section 3.3. Electricity mobility options must be proposed in order to reduce the emission of greenhouse gases.

### 5.4 Discussion

The modeling horizon analyzed is 17-year in three scenarios: 2025, 2030 and 2040 from the year 2023. The investigation identifies potential problems that should not be dismissed, the deferral investment can cause problems in the power system operation. Nowadays, Costa Rica Power System has enough capacity to supply short-term demand, however, future requirement (current demand + electric vehicles) must be supplied with PV and Wind generation and storage [51]. In future works, the impact of the integration of renewable resources in the transmission and distribution grid will be analyzed.

The simulation was performed to the transmission grid and the voltage profiles are analyzed on the high (230 and ) and medium voltage (13.8, 24.9 and ) sides. Transmission voltage levels of , no loadability problems are observed and the voltage profiles remain stable until 2040. However, the  side shows problems due to a large number of loads are concentrated in this voltage level.

In the 15-year horizon, this power grid requires greater investment to increase power transfer capacities and avoid system collapses. The demand growth observed in Table 6 must be planned and expanded by a large investment in electrical infrastructure.

The load modeled (*current load* + *electric vehicles*) is considered in the medium voltage level of the power transformers as mentioned in section 4. No loadability problems are observed until the year 2030 however, loadability condition must be considered and estimated to establish the health index of power transformers. Nowadays, current transformers capacities are 30/ but, these capacities must be reviewed in the future due to loadability after 2030.

A significant rise in the number of vehicles on the road will lead to an increase in the load placed on power system components such as transmission lines, transformers, and generators. Consequently, without adequate investments, the power system will experience overloads, resulting in voltage profiles shifting towards lower levels as the vehicle electrification level continues to grow over time. In order to mitigate the impact on load capacity caused by the high penetration of electric vehicles in the short term, it is imperative to invest in electrical infrastructure, specifically in generation, transmission, and distribution.

The Costa Rican power system exhibits two distinct peaks in its daily demand, which can give rise to issues during those times. Overloaded lines or other elements can result in losses due to voltage drops. Furthermore, specific areas with high purchasing power may experience voltage problems and instabilities due to the high penetration of EVs. Consequently, it is necessary to maintain high levels of reactive power support under conditions of high load integration.

Distribution systems in Costa Rica face challenges, including losses that currently stand at approximately 11.6% and could increase in the future if investments are not made. As a short-term solution, increasing the integration of distributed generation into distribution networks can enhance the capacities and availability of active and reactive power.

This approach becomes particularly important given the growing number of EVs, as distributed generation can help offset the demand at specific points. Consequently, important factors to consider include line overloads and voltage levels resulting from the high penetration of EVs. Finally, the proposed methodology can be implemented at the regional level in Central America, as the networks in this area exhibit high levels of losses compared to the electrical system of Costa Rica.

The study acknowledges several limitations, including the limited availability of data required to use a more complex prediction model capable of considering multiple variables in estimating the penetration of electric vehicles in the country.

Furthermore, the lack of extensive information on population consumption habits prevents the generation of a model that accurately represents real-world behavior. However, the scenario proposed in this study considers current people's electricity usage patterns.

Also, another limitation was that the developed methodology was modeled using the power transmission grid therefore it does not consider charging stations. The lack of information about the charging stations prevents the analysis from estimating the effect on the distribution grid, especially in low voltage.

Future investigations take account of the effect on distribution grid. The investigation focused on analyzing the voltage profiles and demand behavior under extreme operating conditions. It evaluated the loadability and it identified potential problems in the power grid.

## 6 Conclusions and policy implications

This work applies a methodology to assess the impact of electric vehicles on the power transmission grid of the Costa Rica Power System. It takes into account scenarios of penetration, preferences and consumption habits of users, as well as the expected growth of vehicle fleets. The development of the methodology and the analyzed case study allow for the following conclusion:

a. Having a good methodology when analyzing the effects of electric vehicles in a power system is important because ensures that the analysis is conducted in a systematic and transparent way, which helps to identify the key elements and factors that influence the system's performance.
b. Power flows analysis is in 24-hour profiles and it analyzes the loadability of the power system to obtain the voltages and demand. It estimates the transformer and transmission lines loadability analysis under extreme operating conditions. Problems in the electrical infrastructure are found starting in the year 2030. The results in 2040 highlight a major problem as the system is found to be insufficient in incorporating the massive increase in electric vehicles.
c. According to the analyzed models, between 11 and 13 hours there will be a higher integration of vehicles; however, these are hours when the countries in the region can achieve higher levels of solar generation. This would be an alternative for residential, commercial, or any other areas of interest charging stations to take advantage of that energy and smooth out the demand curve.
d. Transmission grids analysis considering the integration of electric vehicles and solar generation has been done by analyzing the loadability and voltage, but it is necessary to evaluate stability due to loadability. However, good management in the distribution networks of solar generation, electric vehicles can improve the performance of power transmission.
e. It is necessary to propose investments in electrical infrastructure of transmission and generation to meet future energy requirements. This investment should be driven by public policies to integrate more distributed energy resources however, the transmission infrastructure must be strengthened.
f. According to established public policies, the actual number of electric vehicles can vary greatly, so it is necessary to take into account the growth of vehicles together with the electric infrastructure in order to analyze changes and improvements to the system.
g. Finally, it's noteworthy that the information in Table 6 presents the minimum and maximum limits. Therefore, the system's predictions and analysis results depict extreme potential scenarios. Consequently, it's anticipated that the system's performance will fall within these demand values.

It is important to point out the necessity of analyzing a broader range of scenarios that consider more variability in parameters, particularly user charging behaviors. By doing so, decision-makers can anticipate and plan for the challenges and opportunities that come with the massive introduction of EVs in a country's power grid.

## CRediT authorship contribution statement

**Gustavo Adolfo Gómez-Ramírez:** Writing – review & editing, Writing – original draft, Methodology, Investigation, Formal analysis, Data curation, Conceptualization. **Rebeca Solis-Ortega:** Writing – review & editing, Writing – original draft, Methodology, Investigation, Formal analysis, Data curation, Conceptualization. **Luis Alberto Ross-Lépiz:** Visualization, Data curation.

## Declaration of Competing Interest

The authors declare that they have no known competing financial interests or personal relationships that could have appeared to influence the work reported in this paper.

## Data Availability

All data will be provided upon request.
